# A drug repurposing screen identifies decitabine as an HSV-1 antiviral

**DOI:** 10.1128/spectrum.01754-24

**Published:** 2024-09-17

**Authors:** Laura Bautista, Cody Sirimanotham, Jason Espinoza, Dillon Cheng, Savaş Tay, Nir Drayman

**Affiliations:** 1 The Department of Molecular Biology and Biochemistry, The University of California Irvine, Irvine, California, USA; 2 Pritzker School of Molecular Engineering, The University of Chicago, Chicago, Illinois, USA; 3 The Department of Microbiology and Molecular Genetics, The University of California Irvine, Irvine, California, USA; 4 The Center for Virus Research, The University of California Irvine, Irvine, California, USA; 5 The Center for Complex Biological Systems, The University of California Irvine, Irvine, California, USA; Barnard College, Columbia University, New York, New York, USA

**Keywords:** herpes simplex virus, antiviral agents, decitabine, lethal mutagenesis

## Abstract

Herpes simplex virus type 1 (HSV-1) is a highly prevalent human pathogen that causes a range of clinical manifestations, including oral and genital herpes, keratitis, encephalitis, and disseminated neonatal disease. Despite its significant health and economic burden, there is currently only a handful of approved antiviral drugs to treat HSV-1 infection. Acyclovir and its analogs are the first-line treatment, but resistance often arises during prolonged treatment periods, such as in immunocompromised patients. Therefore, there is a critical need to identify novel antiviral agents against HSV-1. Here, we performed a drug repurposing screen, testing the ability of 1,900 safe-in-human drugs to inhibit HSV-1 infection *in vitro*. The screen identified decitabine, a cytidine analog that is used to treat myelodysplastic syndromes and acute myeloid leukemia, as a potent anti-HSV-1 agent. We show that decitabine is effective in inhibiting HSV-1 infection in multiple cell types, including human keratinocytes, that it synergizes with acyclovir, and acyclovir-resistant HSV-1 is still sensitive to decitabine. We further show that decitabine causes G > C and C > G transversions across the viral genome, suggesting it exerts its antiviral activity by lethal mutagenesis, although a role for decitabine’s known targets, DNA methyl-transferases, has not been ruled out.

**IMPORTANCE:**

Herpes simplex virus type 1 (HSV-1) is a prevalent human pathogen with a limited arsenal of antiviral agents, resistance to which can often develop during prolonged treatment, such as in the case of immunocompromised individuals. Development of novel antiviral agents is a costly and prolonged process, making new antivirals few and far between. Here, we employed an approach called drug repurposing to investigate the potential anti-HSV-1 activity of drugs that are known to be safe in humans, shortening the process of drug development considerably. We identified a nucleoside analog named decitabine as a potent anti-HSV-1 agent in cell culture and investigated its mechanism of action. Decitabine synergizes with the current anti herpetic acyclovir and increases the rate of mutations in the viral genome. Thus, decitabine is an attractive candidate for future studies in animal models to inform its possible application as a novel HSV-1 therapy.

## INTRODUCTION

Herpes simplex virus type 1 (HSV-1) is a highly prevalent human pathogen, estimated to infect around two-thirds of the global population (1). While most infected individuals do not experience any symptoms, in about 20% of cases, reactivation results in blisters, ulcers, or cold sores around the mouth (2). More rarely, HSV-1 can cause more severe disease, including eczema herpeticum, herpes stromal keratitis, disseminated disease in neonates, meningitis, and encephalitis (2). Thus, HSV-1 is a major cause of morbidity in the human population.

Currently, available antiviral drugs can suppress lytic infection in the epithelia, lessening symptoms, but they do not eliminate the latent reservoir. All currently approved anti-herpetic drugs target the viral DNA replication step by interfering with the activity of the viral DNA polymerase (3). These include acyclovir and its analogs, the recommended first-line treatments, as well as cidofovir and foscarnet. Viral resistance to these drugs is easily acquired by mutations in the viral thymidine kinase or DNA polymerase and is a major issue in treating immunocompromised patients, where the prevalence of acyclovir resistance is estimated to range from 5% to 30% (4). Thus, there is a critical need to identify antivirals with different mechanisms of action.

**Fig 1:**
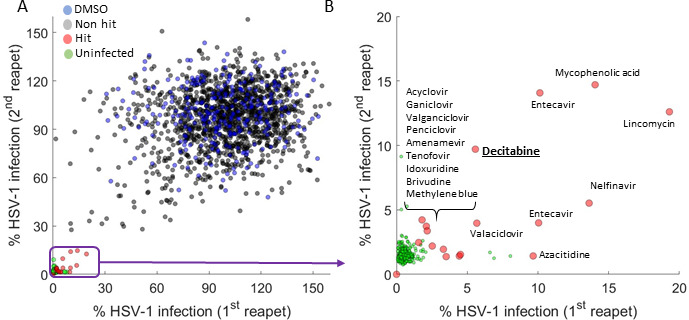
A drug repurposing screen for HSV-1 identified decitabine as a potential anti-herpetic. (**A**) One thousand nine hundred safe-in-human drugs were tested in duplicate for their ability to inhibit HSV-1 infection of A549 cells at 10 µM. Each dot is a single drug. Shown are drugs that did not perturb cell growth (>90%). Drugs are color-coded based on their result in the screen: blue = DMSO control, gray = non-hits (HSV-1 infection reduced by less than 80%), red = hits (HSV-1 infection reduced by 80% or more), and green (uninfected controls). (**B**) Zoom in on the bottom left corner of (**A**), showing the hits from the screen.

Here, we report on a drug repurposing screen, testing the effect of 1,900 safe-in-human drugs on HSV-1 infection *in vitro*, which identified decitabine as a potent inhibitor of HSV-1 infection (Fig. 1). Decitabine is a cytidine analog that is currently used to treat myelodysplastic syndromes and acute myeloid leukemia. It acts by inhibiting DNA methyl-transferases (DNMTs), leading to DNA hypo-methylation and the re-expression of silenced tumor suppressor genes (5). To the best of our knowledge, decitabine has not been previously shown to possess antiviral activity against HSV-1, although it was shown to inhibit infection by another alpha-herpesvirus, Equid Herpesvirus 1 (6), as well as the retroviruses HIV-1 (7, 8), and Feline leukemia virus (9).

We investigated the antiviral properties of decitabine *in vitro* and found that it inhibits HSV-1 infection in clinically relevant concentrations in multiple cell lines, that it is effective against acyclovir-resistant HSV-1, and that it synergizes with acyclovir. We applied an experimental evolution approach to raise decitabine-resistant mutants and found that resistance can indeed be selected for. Decitabine acted as a viral mutagen, causing high rates of transversions across the viral genome, hindering the identification of a single causative-resistant mutant. Decitabine-resistant isolates shared mutations in 14 viral genes, including many that participate in viral genome replication.

While our results are limited to *in vitro* studies of decitabine activity, we conclude that the use of decitabine, or its future derivatives, as a potential HSV-1 therapeutic warrants further investigation.

## RESULTS

### A drug repurposing screen identifies decitabine as an antiviral agent against HSV-1

We began by screening a library of 1,900 safe-in-human drugs for their ability to inhibit HSV-1 infection of A549 cells. We used cells expressing H2B-RFP and a virus expressing ICP4-YFP, which allowed for simple quantification of cell number (to estimate drug effect on cell growth) and viral infection (to determine antiviral effect). Following plating in 384-well plates, the cells were infected at an Multiplicity of infection (MOI) of 0.1, incubated with the virus for 1 hour, and then drugs were added to a final concentration of 10 µM. The plates were incubated for 2 days and scanned to quantify the fluorescence signals. The screen was performed twice, and the data were analyzed to determine which drugs inhibited HSV-1 infection with no or minimal impact on cell growth (Fig. 1; Table S1).

Figure 1A shows the effect of the screened drugs on HSV-1 infection in the two replicates. As expected, the majority of drugs did not have an effect on HSV-1 infection while known anti-herpetics such as acyclovir showed ~95% (20-fold) inhibition of infection. We defined as “hits” drugs that did not affect cell growth (>90% compared to vehicle-treated cells) while reducing viral infection by 80% (fivefold) or more in both replicates (Fig. 1B; Table S1). A total of 18 drugs met these criteria, most of which are known anti-herpetic drugs from the acyclovir family. We decided to follow up on one of these hits, decitabine, as it rescued cell growth in the presence of HSV-1 and was recently described to possess an antiviral activity against a related virus — equid herpesvirus 1 (6).

### Decitabine inhibits HSV-1 infection in multiple cell types

**Fig 2:**
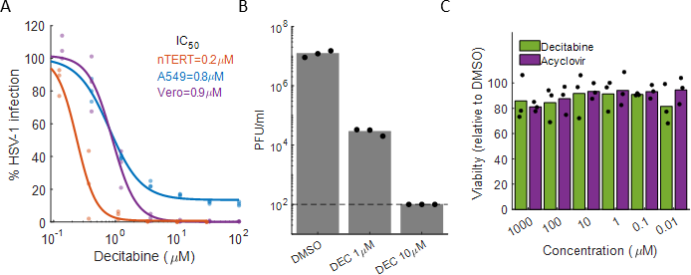
Decitabine inhibits HSV-1 infection in multiple cell lines. (**A**) Dose-response analysis of decitabine inhibition of HSV-1 in nTERT (immortalized human keratinocytes, orange), A549 (blue), and Vero (purple). Dots are individual biological repeats (*n* = 3), and lines are sigmoid curves fitted to the data using Matlab. IC_50_ values are the decitabine concentration needed to achieve 50% HSV-1 infection inhibition. (**B**) Vero cells were infected with HSV-1 at an MOI of 0.1 and treated with 0–10 μM of decitabine for 2 days. Supernatants were collected and assayed for viable progeny by the plaque assay. Dots are individual biological repeats (*n* = 3), and bars represent the mean. (**C**) Effect of decitabine (green) and acyclovir (purple) on cell viability. Dots are individual biological repeats (*n* = 3), and bars represent the mean. Decitabine- and acyclovir-treated cells were normalized to DMSO-treated cells.

Our initial screen was conducted at a single dose of 10 µM in A549 cells, a human lung cancer cell line that is permissive for HSV-1 infection and is commonly used in the field (10–12). To validate the screen’s results and assess the potency of decitabine’s antiviral activity, we conducted dose-response analyses in two cell lines commonly used to study HSV-1 infection (Vero and A549), as well as the more physiologically relevant N/TERT-2 cell line, immortalized human keratinocytes (13) (Fig. 2A). Decitabine was effective in inhibiting HSV-1 in all tested cell types, with IC_50_ values ranging from 200 to 900 nM. We further tested the ability of decitabine to inhibit progeny production. Vero cells were infected with HSV-1 at an MOI of 0.1 and treated with DMSO or 1 µM or 10 µM of decitabine for 2 days. The supernatant of the infected cells was then collected and titrated using the plaque assay. The results (Fig. 2B) show that treatment with 1 µM of decitabine led to ~500-fold reduction in progeny formation and 10 µM reduced progeny formation below the limit of detection.

Under these experimental conditions, we did not observe any effect of decitabine on cellular growth in concentrations up to 1 mM (Fig. 2C). Of note, the maximal tolerated dose of decitabine in humans leads to plasma concentration of 1,700 nM (14) and the usual clinical dose to a concentration of 650 nM (15). We concluded that decitabine is a potent inhibitor of HSV-1 infection *in vitro*.

### Decitabine synergizes with acyclovir and is effective against acyclovir-resistant HSV-1

**Fig 3:**
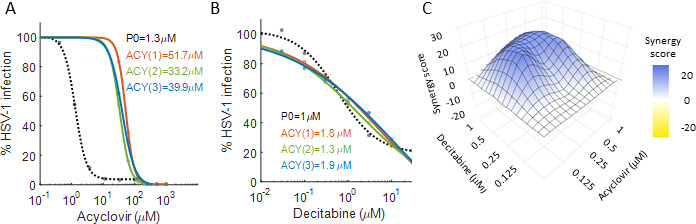
Decitabine is effective against acyclovir-resistant HSV-1 and synergizes with it. (**A and B**) Dose-response analysis of acyclovir (**A**) and decitabine (**B**) inhibition of parental HSV-1 (P0, black) and acyclovir-resistant HSV-1 [ACY (1–3), orange, green, and blue]. Dots are individual measurements (*n* = 1), and lines are sigmoid fits using Matlab. Numbers in legend are the IC_50_ values. (**C**) Synergy plot of combination therapy of acyclovir and decitabine. The x and y axes are the drug concentrations, and the z-axis denotes the synergy score calculated by SynergyFinder. Plotted is the mean synergy score obtained from three independent biological repeats.

As both decitabine and acyclovir are nucleoside analogs, we next assessed whether decitabine’s mechanism of action is distinct from that of acyclovir. To do so, we infected Vero cells with either our parental HSV-1 or three acyclovir-resistant HSV-1 strains derived in our lab and determined the IC_50_ values of acyclovir and decitabine against each strain (Fig. 3A and B). The results show that decitabine is equally effective against the parental strain and the acyclovir-resistant mutants, suggesting it works by a distinct mechanism of action.

As decitabine and acyclovir seem to work by distinct mechanisms, we hypothesized that a combination therapy with both agents should lead to additivity or synergy in their antiviral activity. To test this, cells were infected with HSV-1 at an MOI of 0.1 and treated with 0–1 µM of acyclovir, decitabine, or their combinations (25 conditions in total), in triplicates. The percentage of infected cells was determined 48 hours later by fluorescent microscopy and normalized to vehicle-treated cells. SynergyFinder (16) was used to analyze the data and determine if the combination therapy was synergistic, additive, or antagonistic. As seen in Fig. 3C, the combination therapy of decitabine and acyclovir shows clear synergism (mean synergy score = 10.3, HSA model, *P*-value = 0.006). Thus, despite both drugs being nucleoside analogs, decitabine and acyclovir appear to work by distinct and synergistic mechanisms.

### Experimental evolution leads to HSV-1 acquisition of resistance to decitabine

**Fig 4:**
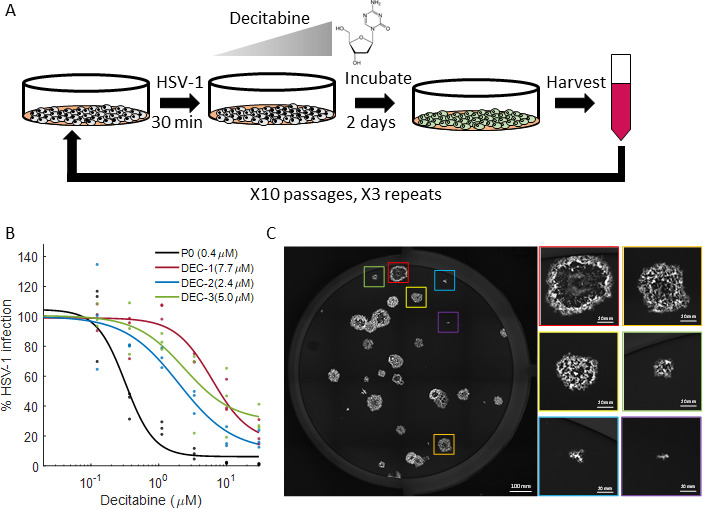
Experimental evolution of HSV-1 in the presence of decitabine. (**A**) Schematic of the experimental evolution design. (**B**) Dose-response analysis of the parental (P0, black) and evolved strains (DEC1-3, red, blue, and green). Dots are independent measurements (*n* = 3), and lines are sigmoid fits using Matlab. Numbers in legend are the IC_50_ values. (**C**) A representative image of plaques formed by decitabine-resistant mutants. The large image shows the YFP-ICP4 of an entire well (bar = 100 mm), and zoomed boxes show six individual plaques at the same magnification (bar = 20 mm).

Given the high potency of decitabine against HSV-1, we hypothesized that it might work as a direct-acting antiviral. We set to determine if HSV-1 can acquire resistance to decitabine treatment through experimental evolution (Fig. 4A). We chose to perform this in Vero cells, as these are the cells used to grow HSV-1 stocks, and we wanted to avoid any mutations that are the result of adaptation to a different cell type. We serially passaged our parental virus in sub-lethal concentrations of decitabine, starting from a quarter of the IC_50_ value and using a low MOI of 0.01, in three independent experiments. After each round of selection, progeny viruses were collected and titered to ensure we maintained an MOI of 0.01 throughout the experiment. We allowed the virus to evolve under these conditions for 10 passages, with increasing concentrations of decitabine. Following the 10th passage, we compared the evolved and parental strains (Fig. 4B). We found that the evolved strains had increased IC50 values, ranging from 5- to 19-fold higher, suggesting that decitabine functions as a direct-acting antiviral against HSV-1.

### Decitabine-resistant HSV-1 exhibits high phenotypic and genetic variability

**Fig 5:**
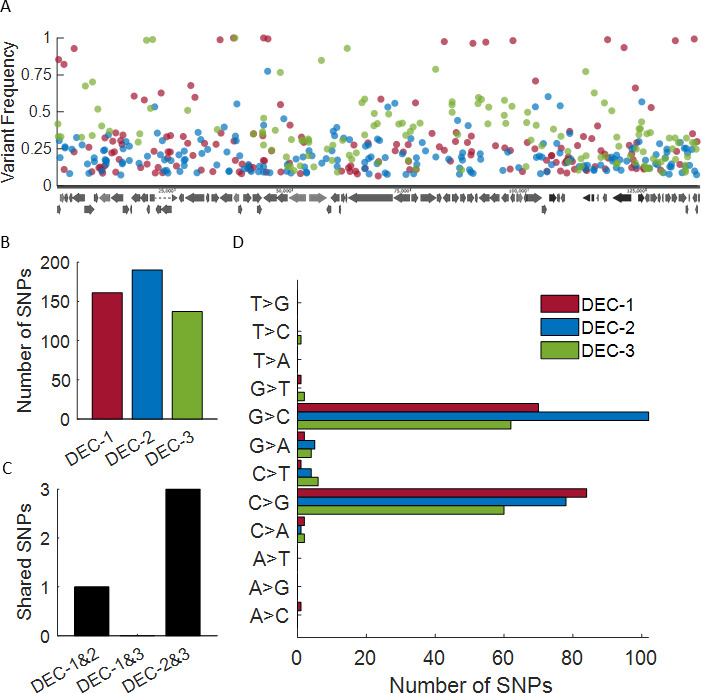
Decitabine-resistant HSV-1 shows high genetic variability. (**A**) Map of SNPs identified in the HSV-1 genome. X-axis is the genome coordinate, and y-axis is the variant frequency. Dots are individual SNPs (DEC-1 = red, DEC-2 = blue, and DEC-3 = green). (**B**) Total number of SNPs detected in the decitabine-resistant mutants. (**C**) Total number of SNPs shared for each of the two mutants. (**D**) Analysis of detected SNPs by mutation type.

During the titration of our decitabine-resistant strains, we noticed a high degree of variability in plaque morphology (Fig. 4C), which we never saw with the parental strain or acyclovir-resistant strains, suggesting our decitabine-resistant strains were a mix of variable progeny. We extracted and sequenced the viral DNA from our parental and decitabine-resistant strains, hoping to uncover the viral target of decitabine. Instead, we observed a high degree of genetic variability that evolved during decitabine treatment. While the parental strain had 16 single nucleotide polymorphisms (SNPs) compared to the reference strain 17 genome, the decitabine-resistant mutants had 139–195 SNPs of variable frequency and were scattered across the entire genome with little concordance between the three resistant strains (Fig. 5A through C; Table S2). Of note, this high degree of genetic and phenotypic variability was not the result of extensive passaging *in vitro*, as HSV-1 passaged for 10 passages in increasing concentrations of another hit from this screen, mycophenolic acid, had very few SNPs compared to the parental strain (Table S3) and did not show variability in plaque morphology (Fig. S1).

Analysis of the type of SNPs observed revealed that 74%–96% were G > C or C > G transversions (Fig. 5D), in agreement with decitabine’s known activity as a lethal mutagen against HIV-1 (17, 18), suggesting decitabine exerts its antiviral activity through lethal mutagenesis of HSV-1.

### Genetic mapping of decitabine resistance

**Fig 6:**
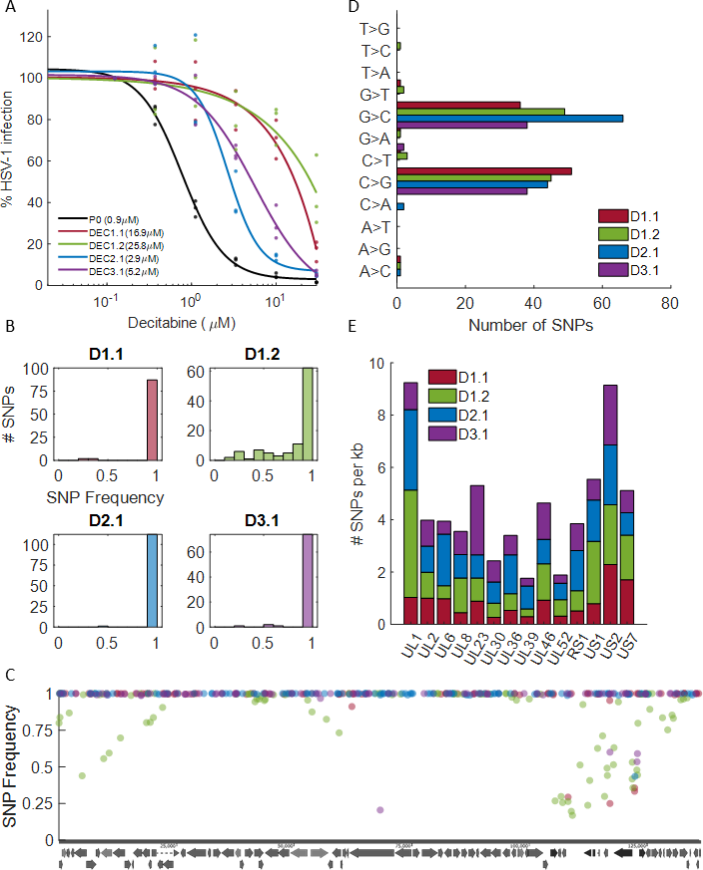
Genetic analysis of plaque purified decitabine-resistant HSV-1. (**A**) Dose-response analysis of decitabine inhibition of parental HSV-1 (P0, black) and four plaque-purified decitabine-resistant isolates (DEC1.1 = red, DEC1.2 = green, DEC2.1 = blue, and DEC3.1 = purple). Dots are individual biological repeats (*n* = 3), and lines are sigmoid fits. Numbers in figure legend are IC_50_ values. (**B**) Histograms of the SNP frequencies for the four isolates. (**C**) Position and SNP frequency of the detected SNPs across all four isolates. Dots are individual SNPs, color-coded as in (**A**). (**D**) Analysis of detected SNPs by mutation type. (**E**) Stacked bar plot, showing the number of SNPs per kb detected in each isolate in the 14 viral genes that were common to all isolates.

As it became clear that our decitabine-resistant strains were a mix of highly mutagenized progeny, we decided to plaque purify and sequence individual isolates. Four individual plaques from the three resistant strains were isolated and subjected to three rounds of plaque purification in the absence of decitabine to prevent additional mutagenesis. The resulting isolates were assayed for decitabine resistance (Fig. 6A), and all isolates exhibited increased IC50 values compared to the parental strain (3- to 28-fold).

We extracted and sequenced viral DNA from the four isolates and confirmed their clonal nature as the vast majority of SNPs were fixed, with allele frequency close to 1 (Fig. 6B). For each isolate, we again observed a high number of SNPs (78–113) that were scattered across the entire genome (Fig. 6C) and were mostly G > C and C > G transversions (Fig. 6D). As before, we did not identify any single SNP that was shared among all isolates (Table S4).

We next analyzed the SNPs at the level of viral genes and found 14 genes that had at least one SNP in all four isolates (Fig. 6E; Table S5). Some of these are structural components of the virion and are unlikely to be the source of decitabine resistance. However, many of the commonly mutated viral genes are involved in nucleotide synthesis and viral DNA replication, which could potentially explain decitabine resistant. These included UL2 (uracil-DNA glycosylase), UL8 and UL52 (helicase-primase), UL23 (thymidine kinase), UL39 (ribonucleotide reductase), and UL30 (DNA polymerase). Further work is needed to test the role of these SNPs in HSV-1 resistance to decitabine.

## DISCUSSION

In this study, we conducted a drug repurposing screen using a library of 1,900 safe-in-human drugs and identified decitabine, a cytidine analog, as a potent antiviral against HSV-1. Similar findings have been reported in a previous screen against a related non-human alpha herpesvirus, EHV-1, where decitabine was effective alone and in combination with acyclovir (6). Our data suggest that decitabine acts by inducing lethal mutagenesis. Interestingly, while we observed similar rates of G > C and C > G transversions during decitabine treatment, another study has observed mainly G > C transversions during HIV-1 infection (18), suggesting a similar yet distinct mechanism of action for decitabine against herpesviruses and retroviruses.

Decitabine is currently used for the treatment of myelodysplastic syndromes and acute myeloid leukemia. In that context, decitabine works by incorporating into replicating DNA and covalently binding to DNMTs, trapping them, and causing global hypomethylation (5). Whether inhibition of host DNMTs contributes to the antiviral activity of decitabine against HSV-1 is currently unclear. On the one hand, the viral DNA is already unmethylated (19), and a recent genome-wide CRISPR screen did not find DNMTs to be required for HSV-1 infection (20). On the other hand, a previous manuscript provided compelling evidence for a role for DNMT3A in HSV-1 infection (21). Further study is needed to elucidate the role of DNMTs inhibition in decitabine’s anti-herpetic activity.

This mechanism of decitabine inhibition of HSV-1 is different from the existing suite of anti-herpetic drugs, such as acyclovir and its analogs, which act as chain terminators during HSV-1 genome replication. Importantly, decitabine was effective against acyclovir-resistant HSV-1 strains, and a combination therapy of acyclovir and decitabine was synergistic. This further underscores decitabine’s unique mode of action and potential as a valuable addition to the current antiviral arsenal.

Resistance studies through experimental evolution revealed that HSV-1 can indeed develop resistance to decitabine. The decitabine-resistant strains exhibited large morphological changes, in agreement with the large increase in the number of single nucleotide polymorphisms. This high genetic variability made pinpointing the specific genetic determinants of resistance highly challenging.

Despite identifying 14 viral genes with recurring mutations in decitabine-resistant isolates, further functional studies are required to elucidate their roles in conferring resistance. Many of these genes are involved in nucleotide synthesis and viral DNA replication, presenting potential targets for future investigations. One promising candidate is the viral thymidine kinase, which might be able to better phosphorylate decitabine than its human counterpart. Understanding the interplay between these genetic alterations and decitabine resistance is crucial for optimizing its antiviral application and developing derivatives with improved efficacy and resistance profiles.

This study has several limitations. First, decitabine’s activity was only tested *in vitro*, using immortalized cell lines. Evaluating decitabine’s antiviral effects in primary cells and animal models will be essential to confirm its therapeutic potential against HSV-1 and support potential clinical trials. Second, while our results suggest decitabine’s is a direct-acting antiviral, we did not rule out the potential role of DNMTs or other host factors in mediating decitabine’s antiviral properties and cannot definitively prove that the antiviral effect is solely attributed to mutagenesis. Third, previous manuscripts reported that decitabine can enhance replication and oncolytic activity of an oncolytic mutant HSV-1 (22) and induce the reactivation of HSV-2 from latency (23), suggesting decitabine might be better explored as a topical treatment rather than a systemic one. Lastly, the possible mutagenic activity of decitabine on host cells would need to be further assessed.

In conclusion, this study presents decitabine as a promising antiviral candidate against HSV-1. Its unique mechanism of action, efficacy against acyclovir-resistant strains, and potential for synergy with existing antiviral drugs make it a compelling candidate for further development. Future studies should focus on *in vivo* evaluations and further mechanistic elucidations to fully harness decitabine’s therapeutic potential against HSV-1.

## MATERIALS AND METHODS

### Cells

Vero cells (ATCC CCL-81) were a kind gift from Dr. Matthew Weitzman. A549 expressing H2B-mRuby was previously reported (24). Both Vero and A549 were maintained in Dulbecco's Modified Eagle Medium (DMEM) + 10% bovine calf serum. N/TERT-2 cells were a kind gift from Dr. Scott Atwood and were maintained in Ker-SFM media with the recommended supplements (Gibco).

### Viruses

HSV-1 strain 17 expressing YFP-ICP4 has been previously reported (25) and was a kind gift from Dr. Matthew Weitzman. HSV-1 expressing YFP-ICP4 and RFP-VP26 (designated ND02) was created in our lab by recombination of the YFP-ICP4 expressing virus with an RFP-VP26 expressing virus (OK14, a kind gift from Dr. Oren Kobiler) and plaque purified multiple times. The drug screening was performed using the YFP-ICP4 expressing virus. All other experiments were performed with ND02. Viral stocks were grown by infecting Vero cells at a low MOI and harvesting the supernatant several days later. Stocks were titrated by a plaque assay.

### Drug screening

Drug screening was performed as previously described (24). In brief, A549-mRuby cells were plated in 384-well plates at a final volume of 30 µL. The following day, HSV-1 was added to the cells (MOI 0.1, 20 µL volume). Plates were imaged to get an initial cell count, and 50 nL from the Selleck FDA-approved drug library (cat #L1300, Selleck) was added. Plates were then incubated for 2 days at 37°C, 5% CO_2_, and imaged again to determine the drugs effect on cell growth and HSV-1 infection.

### Dose-response analysis

Dose-response analysis was performed by seeding cells in 96-well plates, infecting them with HSV-1 at an MOI of 0.01 and adding serial dilutions of the drug. Two days later, plates were imaged to quantify HSV-1 infection level. Vehicle-only controls were used for normalization. A sigmoid fit was used to extract IC_50_ values using Matlab.

For toxicity measurements, cells were seeded and treated similarly in the absence of viral infection. 4',6-diamidino-2-phenylindole (DAPI) was added to the cells 2 days later and imaged to quantify cell counts.

For synergy assays, cells were seeded and treated as above with various combinations of the two drugs. SynergyFinder (16) was used to calculate the synergy score.

### DNA extraction and sequencing

The supernatant of infected cells was cleared from any intact cells by low-speed centrifugation at 500 g for 10 min at 4°C. The cleared supernatant was subjected to high-speed centrifugation (23,000 g, 2 hours, 4°C) to pellet virions, which were then incubated overnight in 200 µL of lysis buffer (0.4 mg Proteinase K, 0.1M NaCl, and 0.5% SDS in H_2_O). Following the incubation, RNA was degraded by the addition of 1 µL RNAse A and incubating at 56°C for 5 min and 65°C for 30 min. The lysate was then subjected to two rounds of organic solvent extraction, first in Phenol:Chloroform:Isoamyl Alcohol (25:24:1) and then in Chloroform:Isoamyl Alcohol (24:1). DNA was precipitated from the aqueous phase by the addition of ice cold 100% ethanol, which was washed once with 70% ethanol and resuspended in DNAse-free water.

The clean DNA was used for library prep for sequencing according to the manufacturer protocol (NEBNext Ultra II FS DNA Library Prep, cat #E6177S) and sequenced on the NovaSeq6000 platform.

### Variant calling

Raw FASTQ files were trimmed using Trimmomatic-0.39 (PE –phread 33, ILLUMINACLIPadapters/TruSeq3-PE.fa:2:30:10 LEADING:3 TRAILING:3 SLIDINGWINDOW:4:15 MINLEN:36), aligned to a concatenated genome of the Vervet monkey and HSV-1 strain 17 using bwa mem, de-duplicated using Picard MarkDuplicates (REMOVE_DUPLICATES = true), and indexed using samtools. Reads mapped to the HSV-1 genome were extracted using samtools, and variant calling was performed using bcftools (-a FORMAT/DP -q 20 -Q 20) to create a vcf file. Variants supported by less than 20 unique reads were discarded. Vcf files were further analyzed by custom-made Matlab scripts to specifically analyze the frequency, location, and type of SNPs as presented in the manuscript.

## Data Availability

All raw sequencing files were uploaded to SRA under project number 1123273 and are available at this link: https://www.ncbi.nlm.nih.gov/bioproject/1123273. VCF files and Matlab scripts for their analysis are available on GitHub at: https://github.com/nirdrayman/Decitabine.

## SUPPLEMENTAL MATERIAL

Figure S1 spectrum.01754-24-s0001.pdf Plaque morphology of 10-passeged MPA virus.

Table S1 spectrum.01754-24-s0002.xlsx HSV1 drug repurposing screen results.

Table S2 spectrum.01754-24-s0003.xlsx Variants in P10 decitabine mutants.

Table S3 spectrum.01754-24-s0004.xlsx Variants in P10 MPA mutant.

Table S4 spectrum.01754-24-s0005.xlsx Variants in plaque purified decitabine mutants.

Table S5 spectrum.01754-24-s0006.xlsx Variants in plaque-purified decitabine mutants—gene level.

ASM does not own the copyrights to Supplemental Material that may be linked to, or accessed through, an article. The authors have granted ASM a non-exclusive, world-wide license to publish the Supplemental Material files. Please contact the corresponding author directly for reuse.
