# Supplementary material for: A drug repurposing screen identifies decitabine as an HSV-1 antiviral
Source: Microbiol Spectr. 2024 Sep 17;12(11):e01754-24. doi: 10.1128/spectrum.01754-24 (PMC11537057; doi:10.1128/spectrum.01754-24)
Supplement: Figure S1 — Plaque morphology of 10-passeged MPA virus. [file spectrum.01754-24-s0001.pdf]

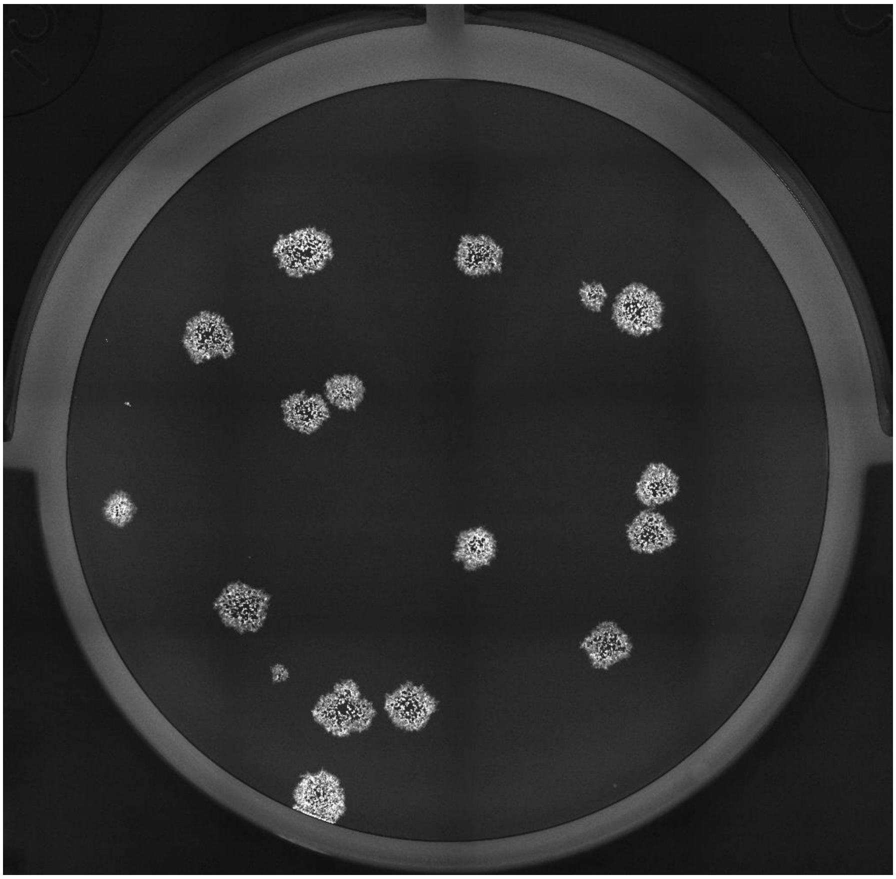

**Figure S1: Experimental evolution of HSV-1 in the presence of mycophenolic acid for 10 passages does not results in variable plaque morphology**
